# Global disparities in access to cancer care

**DOI:** 10.1038/s43856-022-00097-5

**Published:** 2022-04-07

**Authors:** Isabel dos-Santos-Silva, Sudeep Gupta, Jackson Orem, Lawrence N. Shulman

**Affiliations:** 1 Department of Non-Communicable Disease Epidemiology, London School of Hygiene and Tropical Medicine, London, UK; 2 Department of Medical Oncology, Tata Memorial Centre, Mumbai, India; 3 Uganda Cancer Institute, Kampala, Uganda; 4 Center for Global Cancer Medicine, Abramson Cancer Center, University of Pennsylvania, Philadelphia, PA USA

## Abstract

Despite the significant advances made in our understanding of cancer and how to treat it over the last hundred years, there are wide global disparities in access to cancer care and in who gets to benefit from cutting-edge cancer research.

In this Viewpoint, four experts with backgrounds in cancer epidemiology, global health and oncology highlight the scale of the problem and outline their thoughts on steps needed to improve access to quality cancer care in low- and middle-income countries. Strategies to improve resourcing and staffing are identified as important, as well as the need to better coordinate collaborative efforts with high-income countries.

## Isabel dos-Santos-Silva

**Figure:**
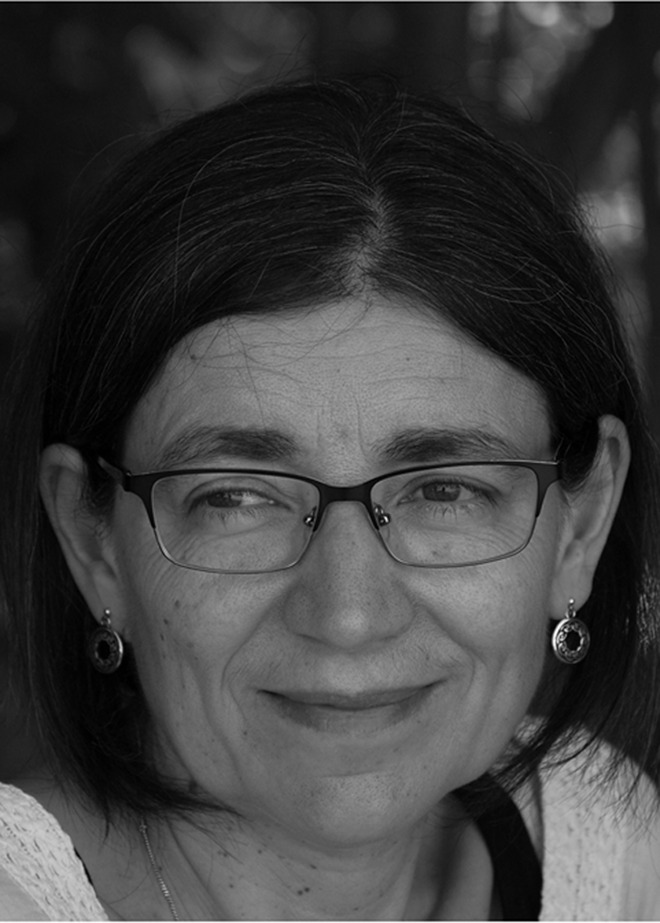
Isabel dos-Santos-Silva

Cancer used to be predominantly a disease of affluent populations, but in recent decades has become a leading cause of morbidity and mortality in low- and middle-income countries (LMICs)^1^. Despite major breakthroughs in cancer care over the last few decades, progress has been uneven, resulting in growing inequalities in access to cancer care between and within countries. Socially disadvantaged population sub-groups defined by demographic and socio-economic status—particularly those living in LMICs—experience poorer health which includes higher proportions being diagnosed at an advanced stage when prognosis is worse, poorer access to effective and affordable treatments, and lower survival rates^2^.

These disparities extend across the whole cancer care continuum, from prevention to diagnosis, treatment, survivorship and end-of-life care. The reasons for this are multifactorial, ranging from contextual (e.g. socio-cultural taboos) to patient-related (e.g. poor cancer awareness, low socio-economic status) and provider-related factors (e.g. weak and fragmented healthcare systems, uneven distribution of resources and services, costs). Furthermore, socio-economically disadvantageous cancer patients in LMICs, and their families, are at risk of impoverishment due to catastrophic out-of-pocket healthcare costs and lost productivity^3^, forcing them to divert resources from other areas, including their children’s education, further trapping them into a cycle of poverty.

The number of newly-diagnosed cancer cases in LMICs is expected to increase substantially in the coming decades due to population growth, rises in life expectancy, and increased exposure to cancer risk factors which historically have been more prevalent in affluent countries, such as tobacco smoking, alcohol consumption, and unhealthy diets. Extending cancer care to millions of people living in LMICs is an urgent health and ethical imperative, particularly as the predicted rise in cancer incidence will aggravate the long-term economic burden of the disease on societies, households and already overburdened health systems, further exacerbating disparities in cancer care. Cancer deaths can be prevented in these settings, and human suffering alleviated, even if there is no access to specialised services or the latest medical diagnostic tools and therapeutics.

Tackling the growing cancer burden in LMICs, whilst achieving equity in care, will require long-term concerted global efforts combined with strong national leadership and locally-tailored culturally-sensitive initiatives. Although increasing numbers of LMICs have developed national cancer care plans over the last decade, many have not dedicated sufficient resources to fund their implementation. Demonstration projects are needed to provide empirical evidence on the best strategies to build sustainable and affordable cancer care in resourced-constrained settings. Nevertheless, in the face of limited resources and competing demands, it is vital to identify priorities to ensure maximum health gains with the rather limited resources available. Properly assessed resource-stratified programmes, in which cancer care strategies are prioritized according to the level of resources available—such as the resource-stratified care guidelines for breast cancer developed by the Breast Health Global Initiative^4^—would help policy makers and health professionals to make the best use of scarce resources. However, any intervention that aims to reduce the overall burden of cancer in a population may inadvertently increase existing disparities in cancer care if disadvantaged population sub-groups are excluded. Providing universal health coverage, a current priority of WHO and a major target of the United Nations Sustainable Development Goals, will be key to achieving equity in access to essential components of cancer care in LMICs, including equitable access to clinically effective, safe and affordable cancer medicines^5^.

## Sudeep Gupta

**Figure:**
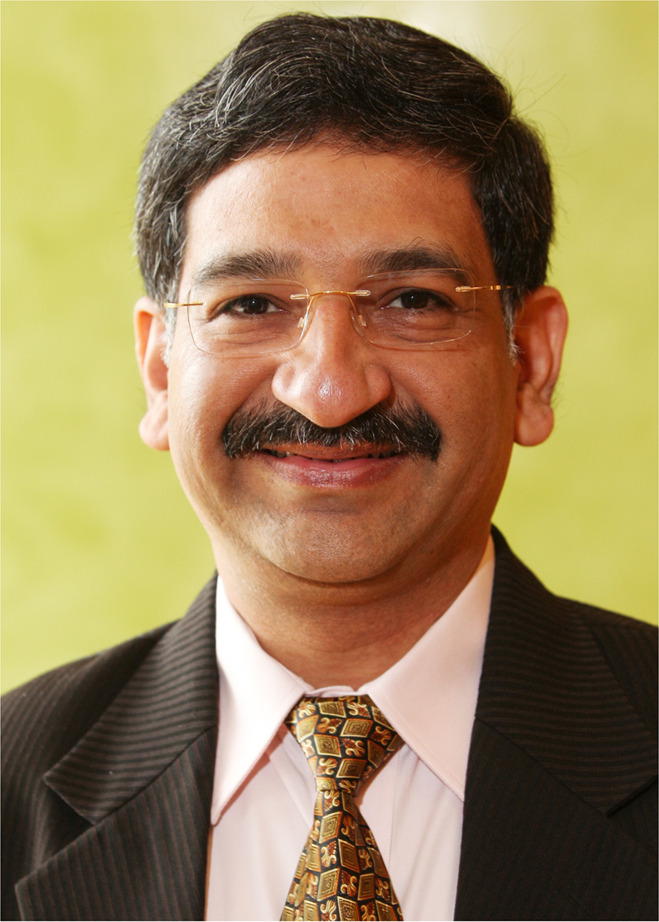
Sudeep Gupta

The modern treatment of almost all cancers requires close collaboration between experts from various disciplines, adequate healthcare infrastructure, and access to an array of systemic therapies and supportive care. There is wide variability in the availability of these resources globally, with significant gaps in many LMICs. The crude and age-adjusted incidence of cancer is lower in LMICs compared with high-income countries (HICs) but their populations are often larger. This means that the absolute numbers of cancer patients—the ‘cancer burden’—is high in LMICs and the majority of the world’s cancer cases and deaths now occur in these countries. Furthermore, many LMICs are expecting a gradual ageing of their population in the coming decades. Cancer incidence and burden, and that of other non-communicable diseases, is likely to increase commensurately. The combination of high cancer and other non-communicable disease burden and inadequate resources to deliver healthcare to large populations in LMICs is a pressing and urgent problem that needs short-, medium- and long-term solutions.

The proportion of gross domestic product that is spent on public health in LMICs is generally lower than that in HICs. The low health expenditure results in inadequate infrastructure at various levels, with underprivileged sections of the population being the most affected. A pertinent example in cancer care is the availability of radiotherapy machines which are vital for management of many cancers, including those that are common in Asia and Africa. In one recent analysis, it was estimated that there was marked worldwide disparity in radiotherapy utilisation rate with low utilisation in many LMICs^6^. The average number of megavoltage radiotherapy machines per 1000 patients requiring radiotherapy was 0–2 in most LMICs compared with 2–5 in HICs. The situation is similar with respect to access to other vital, life-saving cancer treatments, particularly targeted drugs like trastuzumab^7^.

Importantly, resource disparities also exist in relation to the availability of trained personnel to treat cancer. As an example, another recent analysis found wide disparities in the workload of medical oncologists between LMICs and HICs, with high workloads—indicating human resource gaps—in many LMICs^8^. The situation is similar with respect to trained surgeons, pathologists, radiologists, radiation oncologists and others who make up modern multidisciplinary cancer care teams.

The near- and mid-term solutions for some of these constraints include evolving innovative care pathways such as use of acceptable evidence-based, short-course radiotherapy and systemic therapy schedules; creating adequate training opportunities in relevant fields; enabling public-funded medical schools to care for common cancers; and establishment of defined referral pathways for cancers that require complex treatment. The current resource-constrained scenario also means that the primary mechanism for early detection of cancers in many LMICs will be creating awareness and promoting health-seeking behaviour in the population rather than systematic population-based screening with techniques like mammography. The long-term solution will essentially rely on economic development coupled with increasing prioritisation of healthcare at national and regional levels.

## Jackson Orem

**Figure:**
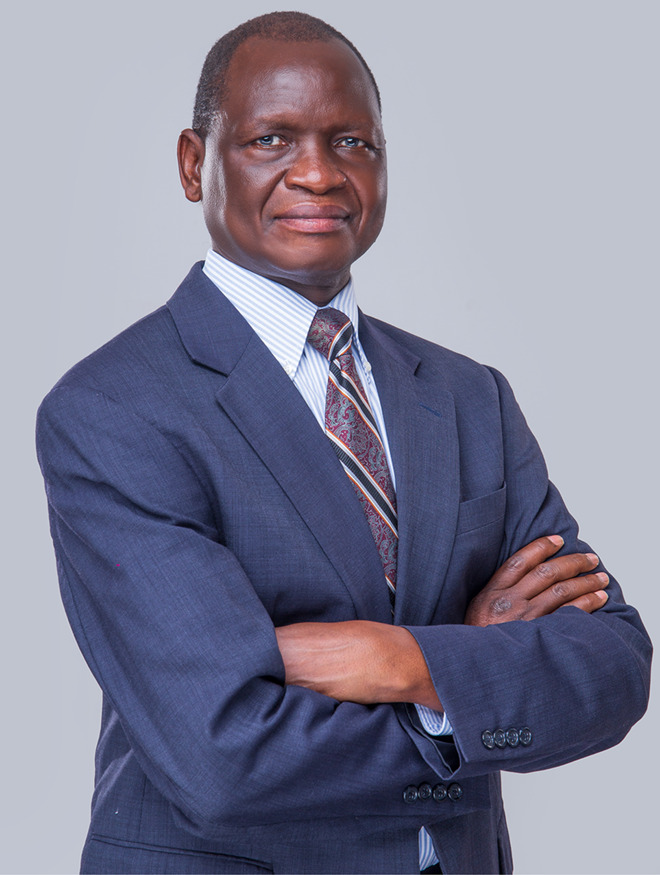
Christine Namulindwa

Cancer care is a complex, constantly evolving and research-driven endeavor. Most LMICs, including those in Africa, would like to establish comprehensive cancer care programs. This is hampered, however, in part by a lack of embedded best practices and supportive public health systems^9^. It is also hampered by a poor appreciation of the importance of cancer research as the key to improving access to cancer care. It is often assumed that research findings obtained in the developed world will automatically be translated and put into practice in LMICs, and that hence there is little need for original research in these countries. This is not the case.

Sub-Saharan Africa has made tremendous contributions to the development of modern oncology practice and science. One such example is the landmark work of Dr. Denis Burkitt, describing Burkitt’s lymphoma in 1958^10^, which drew on observations made in patients in Uganda and was performed in close collaboration with doctors and scientists at Makerere University, Kampala. Both at the time and in subsequent years, African scientists have made key contributions towards understanding the epidemiology, biology and genetics of Burkitt’s lymphoma, as well as its relationship to malaria and Epstein-Barr virus. African researchers have also been pivotal in the development and testing of treatments for Burkitt’s lymphoma, including chemotherapy^11^. However, despite making important contributions to understanding this disease and other cancers, the dividends of cancer research are slow to trickle down to hospitals and cancer patients in Africa, due to the socio-economic inequalities between HICs and LMICs particularly in sub-Saharan Africa.

Drawing on the above in a series of lectures, Dr. Harold Varmus, the past Director of the National Cancer Institute in the US, noted the importance of medical research carried out in developing countries and its contribution to global science^12^. He thought, and rightly so, that research should be an integral part of responses to challenging health situations in developing countries. However, without a specific global research policy, it’s challenging to achieve this objective however well-meaning and well-funded this research is. This is exemplified by the situation with research into HIV-related cancers in sub-Saharan Africa, which to date has lacked gravitas despite HIV being a major driver of malignancies in the region^13^. Although links between HIV and cancer are now better understood, this was not the case at the outset of the HIV pandemic in Africa, and so cancer was not included in major research initiatives such as the Global Fund (www.theglobalfund.org).

Moving forward, research should become an integral part of international responses to health challenges including cancer in LMICs. However, this must be preceded by a global policy alignment with prevailing cancer research strategies and practices in the developed world. This will be a new paradigm which will help to close the equity gap that currently exists in harnessing the benefits of global cancer research to control cancer.

## Lawrence N. Shulman

**Figure:**
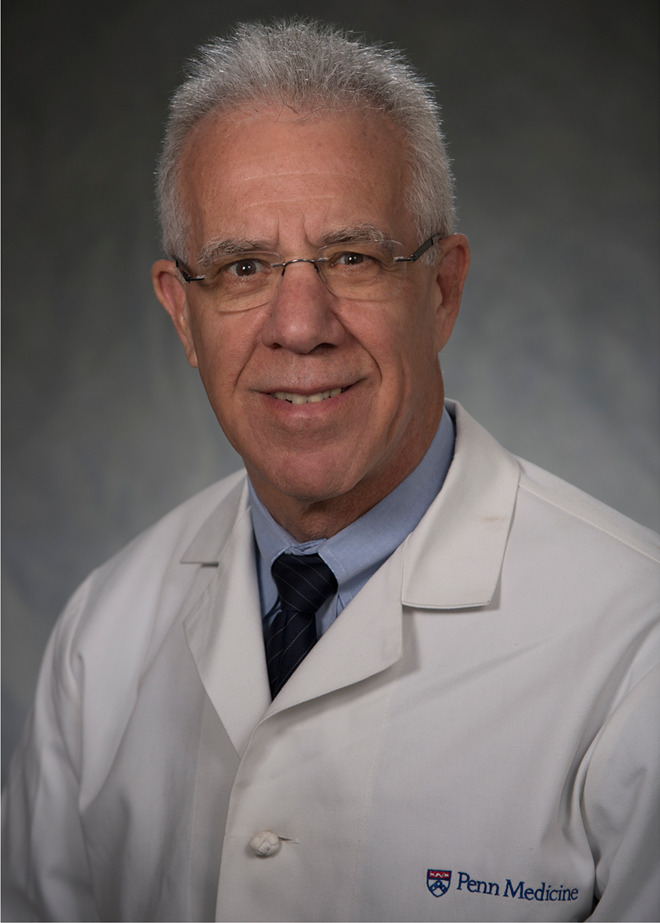
Lawrence N. Shulman

In the last five decades, great progress has been made in the treatment of most cancers, with substantial reductions in cancer morbidity and mortality for patients in HICs. Unfortunately, the benefit of these advances are not yet realized for many patients throughout the globe, particularly those living in low-income countries

Twenty years ago, we developed funding and processes that brought effective therapies for HIV, tuberculosis, and malaria to much of the world. Notably, poverty and geography were largely overcome as barriers to receiving these life-saving treatments. Reductions in global infectious disease mortality have contributed to people living longer, and therefore cancer incidence is rising in all regions of the world. Cancer mortality now far outpaces that from infectious disease, but unlike the heroic efforts to fight the latter, geography and poverty remain deep barriers to access modern, high-quality cancer care.

Cancer is a complex, heterogeneous set of diseases for which provision of high-quality diagnostics and treatments can be challenging even in HICs. However, some have shown that cancer care can safely and effectively be delivered in the most resource-constrained settings, with the result of countless people surviving their disease when previously they would not have. Programs developed to deliver cancer care in these settings must employ well-conceived, structured approaches, which include mechanisms to assure that poverty is not a barrier to access. One must assure that the five Ss are all systematically addressed: staff, stuff, space, systems, and social support. Staff—physicians, nurses, laboratory technicians and other supporting personnel—must be present and trained. The stuff needed for cancer care must be available, including drugs and the equipment necessary to safely deliver them. Space designed for caring for cancer patients which provides a safe inclusive environment is critical. Systems that support the care, avoid medication and reagent stock-outs, reliably provide quality pathology and radiology services, and similar critical components of care are needed. And when caring for the poor, social support is a must. Patients often need transportation, food security, and support to allow them time away from family and work, for them to be able to avail themselves of cancer services. Missing any one of these will result in a weak link in the chain, leading to sub-optimal care and outcomes. It is critical that efforts to provide cancer care in these settings be accompanied by prospective implementation of research to assess the effectiveness and safety of the care provided. Tumor response and toxicity assessment must be routinely measured, along with, most importantly, survival. With this approach, progress can be quantified and remaining gaps elucidated, providing a path towards improved outcomes.

Policy makers, governments, and funders are concerned that cancer care is beyond what can be provided in resource-constrained settings, and there are experiences that confirm poor outcomes when this structured approach, as noted above, is not taken. Cancer therapy is toxic, and unless administered according to recognized guidelines, the possibility remains that more harm will be done than good. It is our global responsibility as a cancer professional community to form true bilateral collaborations between cancer programs in HICs and resource-constrained settings to collaboratively build high-quality cancer care delivery systems in these settings.

Twenty years ago, access to life-saving therapies for HIV, tuberculosis and malaria were collectively agreed to be a human right that should be accessible to the whole of humanity. Currently, much of the world does not have access to life-saving cancer treatment, resulting in millions of preventable deaths every year. We need to change this.
